# Making the impossible possible: Baby KJ and the road map to personalized gene‐editing care

**DOI:** 10.1002/ctm2.70515

**Published:** 2025-10-30

**Authors:** Vanessa Almendro, Sadik H. Kassim

**Affiliations:** ^1^ Danaher Corporation Washington District of Columbia USA

1

Personalized gene editing is rapidly transitioning from concept to clinical practice, marking one of the most significant shifts in contemporary medicine. Over the past decade, the field has advanced from preclinical proof‐of‐concept experiments to the first real‐world demonstrations of patient‐specific, clinically administered genome‐editing therapies. In 2025, this progress culminated in the widely publicized case of “Baby KJ,” a child treated with a bespoke in vivo base‐editing therapy for carbamoyl phosphate synthetase 1 (CPS1) deficiency. This case not only represents a remarkable scientific achievement but also a pivotal clinical milestone, demonstrating that individualized genome editing can be safely and effectively deployed in urgent, life‐threatening conditions.^1^ For clinicians, this case highlights the arrival of a new therapeutic modality with profound implications for diagnosis, care delivery and long‐term disease management in rare genetic disorders.

Carbamoyl phosphate synthetase 1 deficiency is among the most severe urea cycle disorders, typically presenting in the neonatal period with hyperammonemia, neurological compromise and rapid progression to death without liver transplantation. Traditional management relies on dietary restriction, ammonia scavengers and transplantation, but these interventions are palliative or carry high risk rather than curative potential.^2^ In Baby KJ's case, rapid genomic diagnosis revealed a pathogenic single‐nucleotide mutation in the CPS1 gene that was directly amenable to correction through base editing. A multidisciplinary team of clinicians, geneticists and regulatory experts designed an individualized therapy using messenger RNA encoding a base editor and a customized guide RNA, packaged within lipid nanoparticles optimized for liver delivery. The therapeutic rationale was straightforward but powerful: directly correct the underlying mutation in hepatocytes to restore enzyme function, thereby normalizing nitrogen metabolism and preventing further neurotoxic crises.^1^

The treatment journey underscored the complexities of developing personalized medicines. Patient‐specific preclinical assays and genotoxicity evaluations were required within weeks, while cGMP manufacturing for a novel formulation had to be rapidly optimized. Regulatory authorities worked under an emergency investigational new drug framework, allowing expedited review and authorization.^1^ The therapy was delivered through multiple infusions at a critical point when the patient faced imminent metabolic decompensation. Clinicians monitored metabolic parameters, liver enzymes and developmental milestones, while also conducting detailed genomic assays to confirm on‐target editing and rule out off‐target events.

The reported outcomes were encouraging. Following treatment, the patient achieved improved metabolic stability, reduced reliance on conventional therapies and developmental progression that would have been unlikely under standard care. Although long‐term surveillance is essential, the immediate success provided proof of principle that patient‐specific gene editing can alter the natural history of devastating monogenic diseases.

Several technical innovations enabled this success. Base editing, unlike conventional CRISPR‐Cas9, allows for precise nucleotide conversions without generating double‐strand breaks, reducing risks of insertion‐deletion mutations and chromosomal rearrangements.^3^ Lipid nanoparticle delivery of messenger RNA ensured transient expression of the editing machinery, limiting immune complications.^4^ Patient‐specific off‐target profiling, supported by whole‐genome sequencing and computational modelling, further strengthened clinical confidence.^1^ Together, these innovations created a safer and more controlled genetic intervention suitable for acute pediatric care.

The challenge now is to ensure that Baby KJ's case is not remembered as a singular anecdote but as the starting point for scalable and reproducible approaches. The scalability and access drivers for individualized genomic medicine differ fundamentally from those of mass‐market therapeutics.^5^ Proof‐of‐concept demonstrations must evolve into standardized workflows and platforms that can be replicated across patients and indications. At the Children's Hospital of Philadelphia, the team has begun translating the KJ experience into an umbrella platform for liver‐based urea cycle disorders, creating protocols that could support multiple patients through shared infrastructure [personal communication]. Similar precedents exist for personalized genomic medicines: the case of Mila Makovec, who received the custom antisense oligonucleotide (ASO) “milasen” for Batten disease,^6^ demonstrated the feasibility of individualized ASO therapies but also exposed the limits of bespoke approaches without scalable infrastructure.^7^

Achieving scalability in genomic medicine requires several enabling conditions (Figure 1). First, robust patient identification and eligibility systems must be in place, including newborn sequencing programs and rare‐disease networks capable of early detection and referral. Traditional newborn screening (NBS) panels currently identify only 50–60 conditions across most U.S. states, thereby missing thousands of rare monogenic disorders that collectively affect approximately 1 in 300 newborns.^8^ In contrast, pilot newborn genome sequencing initiatives have shown detection rates that are 10–15 times higher than conventional screening, uncovering actionable genetic conditions in 3%–5% of sequenced infants that would have been overlooked by standard panels.^9^ Second, validated design pipelines as platforms must be established to ensure that therapeutic candidates meet safety and efficacy requirements with rapid turnaround.^5^ Third, small‐batch GMP manufacturing capabilities must be built, allowing reproducible production of guides, editors and delivery systems at predictable cost. Finally, reimbursement mechanisms must evolve to recognize these procedures as medically necessary, with clear coding, Medicaid or public payer coverage, and equitable frameworks for access. Once public payers adopt these interventions, the bottleneck will shift from insurance approval to platform capacity and the ability to design, manufacture and deliver patient‐specific therapies in parallel.

**FIGURE 1:**
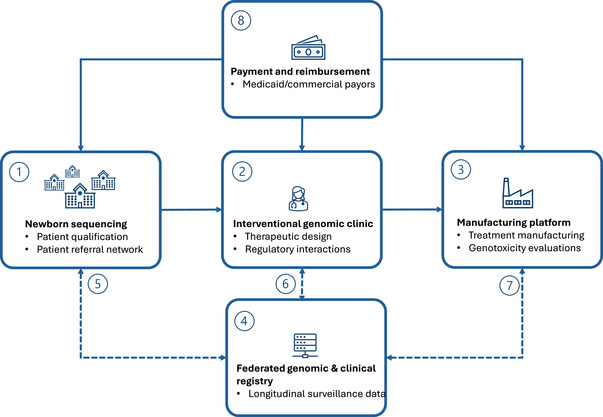
New paradigm for the development of personalized gene editing therapies. Scaling access to personalized gene editing technologies will require (1) streamlining rapid diagnosis of eligible patients at birth through rapid whole‐genome sequencing. Standardization of genomic interrogation and patient diagnosis will be crucial to identify therapeutic options and guide referrals to (2) interventional genomics clinics, where individualized genomic interventions will be designed and delivered to patients. These clinics will lead the design and qualification of specific products in collaboration with (3) central or distributed GMP manufacturing centerrs operating under platform designations. This new ecosystem will depend on (4) a centralized repository for individualized genomic and clinical data that can be accessed in a federated manner to create a knowledge base supporting (5) continuous interpretation of the clinical significance of genomic variants, (6) understanding of patient‐specific traits influencing clinical responses, and (7) evidence‐based design and qualification of patient‐specific interventions. Building a reliable infrastructure for early patient identification, data integration, and longitudinal clinical monitoring will be critical to enable regulatory recognition and (8) sustainable reimbursement for personalized genomic medicine.

Conceptually, personalized genome editing also requires a reframing of how interventions are perceived. Traditional therapies are viewed as treatments: mass‐produced products optimized for large populations. Personalized gene editing more closely resembles an intervention, akin to a surgical procedure. The clinical workflow involves diagnostic work‐up, computational design, interventional administration and longitudinal monitoring. In urea cycle disorders, base editing can be compared with enzyme replacement, scavenger therapy, or transplantation. In neuromuscular disease, antisense oligonucleotides can be weighed against gene replacement or ex vivo cell therapy. This framing emphasizes the procedural nature of genome editing and positions it within interventional medicine rather than pharmaceutical dispensing.

The natural evolution of this thinking is the development of interventional genetic clinics, specialized centes where genome editing becomes a frontline discipline. Unlike conventional genetics clinics, which focus on diagnosis and counseling, these units would function like interventional cardiology suites, equipped to perform direct genetic procedures. Interventional geneticists would be trained to deliver CRISPR‐based editing, base or prime editing, gene replacement therapies and oligonucleotide modulators. Clinics would integrate advanced diagnostics, AI‐driven design, GMP‐adjacent manufacturing and long‐term follow‐up under unified governance. Patients identified at birth could be referred directly for interventions, embedding personalized genome editing into the standard continuum of care and democratizing access.

Data sharing will be also critical to drive scalability. Without interoperable clinical and genomic data infrastructures, each case risks remaining anecdotal. Federated registries and harmonized assay standards will be needed to convert N‐of‐1 experiences into population‐level evidence^10^ (Figure 1), A bottom‐up model could be subsequently deployed to learn from patient‐optimized therapies and generalize to larger cohorts, in contrast with the traditional model of extrapolating population aggregate data to individuals. This will enable regulators to evaluate platforms, clinicians to validate safety and payers to justify reimbursement. Long‐term patient registries will also be critical to monitor durability, immune responses and late‐onset safety issues.

Taken together, these developments signify a broader shift in the pharmaceutical value chain. Traditional drug development has been population‐based, linear and product‐centric. Personalized genome editing is patient‐first, modular and service‐oriented. For industry, this requires modular platforms and flexible GMP capacity. For regulators, it requires new frameworks that assess platforms rather than isolated products. For clinicians, it requires active engagement in diagnosis, intervention, monitoring and data contribution. Precision medicine will transform the value chain, and clinicians cannot remain bystanders. Their participation will determine how successfully these therapies become part of mainstream care and whether access is equitable.

In conclusion, the treatment of Baby KJ with a personalized in vivo gene‐editing therapy is a landmark achievement that demonstrates the feasibility of individualized interventions to rescue patients from fatal trajectories. For clinicians, the message is clear: personalized genome editing is no longer speculative but an emerging therapeutic reality. Hospitals and care teams that prepare for this future—through newborn sequencing, interventional clinic development, standardized protocols and federated data infrastructures—will be at the forefront of delivering life‐changing therapies. The challenge now is to transform single successes into sustainable systems that can serve all patients in need, redefining medicine from the treatment of disease with generalized drugs to direct genetic intervention as a standard component of care.

## CONFLICT OF INTEREST STATEMENT

The authors are employees of Danaher Corporation. Sadik H. Kassim is also a scientific advisor and holds equity in the following genetic medicine companies: Aurora Bio, Koi Bio, nChroma Bio and Profluent.
